# Attention TurkerNeXt: Investigations into Bipolar Disorder Detection Using OCT Images

**DOI:** 10.3390/diagnostics13223422

**Published:** 2023-11-10

**Authors:** Sermal Arslan, Mehmet Kaan Kaya, Burak Tasci, Suheda Kaya, Gulay Tasci, Filiz Ozsoy, Sengul Dogan, Turker Tuncer

**Affiliations:** 1 Universal Eye Clinic, 23119 Elazig, Turkey; drsermal@hotmail.com; 2 Vocational School of Technical Sciences, Firat University, 23119 Elazig, Turkey; 3 Department of Psychiatry, Elazig Fethi Sekin City Hospital, 23100 Elazig, Turkey; suheda_sener@hotmail.com (S.K.); akcagulay01@gmail.com (G.T.); 4 Department of Psychiatry, School of Medicine, Tokat Gaziosmanpasa University, 60100 Tokat, Turkey; filiz.ozsoy@gop.edu.tr; 5 Department of Digital Forensics Engineering, College of Technology, Firat University, 23119 Elazig, Turkey; sdogan@firat.edu.tr (S.D.); turkertuncer@firat.edu.tr (T.T.)

**Keywords:** bipolar disorder, biomarker discovering, OCT image classification, Attention TurkerNeXt

## Abstract

*Background and Aim:* In the era of deep learning, numerous models have emerged in the literature and various application domains. Transformer architectures, particularly, have gained popularity in deep learning, with diverse transformer-based computer vision algorithms. Attention convolutional neural networks (CNNs) have been introduced to enhance image classification capabilities. In this context, we propose a novel attention convolutional model with the primary objective of detecting bipolar disorder using optical coherence tomography (OCT) images. *Materials and Methods:* To facilitate our study, we curated a unique OCT image dataset, initially comprising two distinct cases. For the development of an automated OCT image detection system, we introduce a new attention convolutional neural network named “TurkerNeXt”. This proposed Attention TurkerNeXt encompasses four key modules: (i) the patchify stem block, (ii) the Attention TurkerNeXt block, (iii) the patchify downsampling block, and (iv) the output block. In line with the swin transformer, we employed a patchify operation in this study. The design of the attention block, Attention TurkerNeXt, draws inspiration from ConvNeXt, with an added shortcut operation to mitigate the vanishing gradient problem. The overall architecture is influenced by ResNet18. *Results:* The dataset comprises two distinctive cases: (i) top to bottom and (ii) left to right. Each case contains 987 training and 328 test images. Our newly proposed Attention TurkerNeXt achieved 100% test and validation accuracies for both cases. *Conclusions:* We curated a novel OCT dataset and introduced a new CNN, named TurkerNeXt in this research. Based on the research findings and classification results, our proposed TurkerNeXt model demonstrated excellent classification performance. This investigation distinctly underscores the potential of OCT images as a biomarker for bipolar disorder.

## 1. Introduction

Bipolar disorder (BD) is a psychiatric condition with a global prevalence of approximately 1%, which can significantly impact patients’ social, occupational, and cognitive abilities, and overall quality of life [1]. The exact etiology of bipolar disorder remains uncertain. However, the terms “neuroprogression” and “neurodegeneration” have been emphasized in relation to certain processes underlying this disorder. Neurons can either complete their normal development or remain in an underdeveloped state. Conversely, “neurodegeneration” describes a process in which a normally developed neuron degenerates and loses its function [2]. Neuroprogression processes in bipolar disorder may encompass genetic and epigenetic changes, structural and functional changes in the brain, damage to neuronal circuits, disrupted circadian rhythms, alterations in the immune and hormonal systems, impaired neuronal plasticity, increased cell death, synaptic transmission and signaling issues, activation of neurotoxic mechanisms, and alterations in neurogenesis [3]. Factors contributing to neuroprogression processes in bipolar disorder include the dopaminergic system, inflammatory cytokines, oxidative and nitrosative stress, mitochondrial dysfunction, imbalances in calcium signaling pathways, neuroinflammation, autoimmune responses, disruptions in tryptophan and its metabolites, and irregularities in the hypothalamic–pituitary–adrenal axis [4,5,6]. Brain structural abnormalities have been examined through neuroimaging techniques and associated with numerous susceptible genetic variations in genetic studies [7,8]. Whether bipolar disorder is a neurodegenerative disorder remains a topic of debate. Structural brain changes are considered neurobiological markers of bipolar disorder, since they can be observed even during acute episodes and in remission periods [9]. Recent neuroimaging studies indicate increasing evidence supporting neurodegeneration. Magnetic resonance imaging (MRI) studies reveal structural changes such as increased gray matter in individuals using lithium [10]. Such structural changes can be observed from initial episodes of bipolar disorder [11]. Furthermore, some changes associated with the number of mood episodes, such as reductions in gray matter in the hippocampus, fusiform gyrus, cerebellum, and temporal lobes, may have a progressive nature [12]. Total brain gray matter volume is correlated with the duration of bipolar disorder, and this reduction can be considered an indicator of the neurodegenerative process [13]. Additionally, a relationship has been found between genetic diversity and gray matter deficiency in bipolar disorder, and individuals with these abnormalities may have healthy offspring [14,15]. Numerous studies in the field of neurodegeneration, particularly neuroimaging studies, provide evidence suggesting that bipolar disorder may be a neurodegenerative disorder. The examination of the retina is considered a potential method for early diagnosis of degenerative processes in the central nervous system because retinal nerve axons synapse with many brain regions, making the retina an extension of the central nervous system. Therefore, the retina is often described as a ‘window to the brain’ [16,17]. The inclusion of unmyelinated nerve axons in the retinal nerve fiber layer (RNFL) and its embryological connection to the central nervous system make the RNFL an ideal structure for assessing neurodegeneration. The diagnosis of bipolar disorder is often complex and time-consuming using traditional clinical methods. AI techniques are used in the literature to detect psychiatric diseases [18,19,20,21,22,23,24]. However, in recent years, the combination of optical coherence tomography (OCT) imaging technology and artificial intelligence (AI) techniques has enabled a faster and more accurate diagnosis of this psychiatric disorder. OCT can precisely observe microstructural changes in the retinal nerve fiber layer, while AI algorithms can analyze these data and identify distinctive patterns indicative of bipolar disorder. This article examines the potential of OCT images and AI techniques in the diagnosis of bipolar disorder and discusses how this novel approach could play a role in clinical applications.

### 1.1. Literature Gaps

The literature gaps identified in OCT image classification encompass several key areas. Firstly, a reliance on well-established machine learning or deep learning models has been pervasive among researchers, ensuring high classification performance but constraining the introduction of novelty. Secondly, there is a noticeable scarcity of attention-based models within the existing literature. Lastly, a significant portion of researchers has presented results lacking in explanability, often providing only the classification outcomes.

### 1.2. Motivation

We are now in what is often referred to as the information era. Consequently, many researchers have delved into machine learning to extract significant features from data. In this study, our primary motivation is to introduce a potential new biomarker for diagnosing bipolar disorder: OCT images. We developed a novel convolutional neural network (CNN) named Attention TurkerNeXt to further our research objective. This proposed CNN represents a new iteration of ConvNeXt. From this perspective, our contribution is an original machine-learning model with a focus on attention mechanisms. Additionally, we provide explainable results.

In this research, our primary objective is the detection of bipolar disorder utilizing OCT images. Our approach is grounded in a theory positing that ‘Patterns in optical density across the OCT images may highlight specific biomarkers. Variations in the light absorption characteristics within the retinal layers could signify structural changes associated with bipolar disorder’. To substantiate this theory, we applied the proposed TurkerNeXt to the collected OCT images, revealing a novel biomarker. Our identified biomarker is the OCT image for bipolar disorder, aligning with our central goal of detecting hidden patterns within the data.

We assessed the classification performance of our proposed TurkerNeXt using a collected OCT image dataset. This dataset is divided into two categories: bipolar disorder and control. Furthermore, the dataset encompasses two distinct cases based on eye movement: (i) top to bottom and (ii) left to right. Upon applying the TurkerNeXt to these cases, we obtained specific classification results.

### 1.3. Contributions and Novelties

In terms of contributions and novelties, our research introduced key innovations. Firstly, we curated a new OCT image dataset tailored for detecting bipolar disorder. Secondly, we introduced a novel ConvNeXt variant named “Attention TurkerNeXt.

Our primary aim was to explore the potential of OCT images as a biomarker for bipolar disorder. To achieve this, we collected an OCT image dataset and developed an attention-based ConvNeXt model. The results obtained through Attention TurkerNeXt underscore the viability of OCT images as a biomarker for bipolar disorder detection. Importantly, by leveraging Attention TurkerNeXt, we provided explainable results, positioning our model as an exemplar of explainable artificial intelligence.

## 2. Related Works

In recent years, artificial intelligence techniques have played a significant role in disease detection and diagnostic processes in medicine. In particular, artificial intelligence-based approaches for diagnosing and monitoring eye diseases using OCT images have been extensively researched. Studies in this domain aim to enable faster and more accurate handling of critical health issues such as disease diagnosis and treatment monitoring. While research has been conducted on the use of artificial intelligence for detecting eye diseases from OCT images, there is currently no work related to diagnosing psychiatric disorders using OCT images. Therefore, below is a review of studies in the literature regarding the detection of eye diseases using OCT.

Thomas et al. [25] proposed a method based on contrast enhancement and adaptive noise reduction to extract the retinal pigment epithelium (RPE) layer and determine the drusen height in retinal spectral domain optical coherence tomography (SD-OCT) images. The drusen height was obtained, and the OCT images were classified as either AMD or normal. The classification accuracy in a dataset consisting of 2130 images is 96.66%. Perdomo et al. [26] introduced their deep learning model, OCT-NET, which was developed to classify retinal diseases. Using this model, they classified OCT images of healthy eyes, diabetic retinopathy, diabetic macular edema, and age-related macular degeneration in the SERI-CUHK and A2A SD-OCT datasets. They achieved precision and AUC results of 93% and 86%, respectively, in the SERI+CUHK dataset. In the A2A SD-OCT dataset, they obtained an AUC result of 99.00%. Lee et al. [27] proposed a deep learning-based model for distinguishing between normal and AMD (age-related macular degeneration) based on macular OCT images. Their proposed a modified VGG16 CNN model consisting of 21 layers. The study utilized 52,690 normal macular OCT images and 48,312 AMD macular OCT images. They yielded a disease classification accuracy of 87.63% and an AUC value of 92.77%. Perdomo et al. [28] introduced a CNN-based OCT-NET model to classify OCT volumes automatically to detect diabetic macular edema. With their proposed 12-layer CNN network, they achieved accuracy, sensitivity, and specificity results of 93.75% each. Zhang et al. [29] proposed a more intuitive and robust diagnostic model, considering its self-improvement capability and the ability to assist clinical triage for patients. They used 38,057 OCT images (Drusen, DME, CNV, and Normal) to build and evaluate their model. Their approach utilized a multiscale transfer learning algorithm. Initially, samples were sent to a self-enhancement module for automatic edge detection and improvement. Subsequently, processed data were directed to the image diagnosis module for disease type determination, which improved efficiency and accuracy. The model achieved accuracy, sensitivity, and specificity results of 94.5%, 97.2%, and 97% on an independent test dataset. Abdullahi et al. [30] proposed a 50-layer Deep Residual Neural Network (ResNet50) convolutional neural network for classifying dry and wet AMD. They utilized the KERMANY dataset consisting of 32,931 OCT images for model training. Their proposed method achieved accuracy, specificity, and sensitivity results of 96.56%, 98.20%, and 89.45%, respectively. Saleh et al. [31] aimed to classify patients of choroidal neovascularization, diabetic macular edema, drusen, and normal using optical coherence tomography (OCT) images. They employed two new transfer learning-based techniques, SqueezeNet and InceptionV3Net, to classify retinal disorder. Data segmentation was used to compare two SqueezeNet scenarios consisting of 11,200 OCT images for one and 18,000 for InceptionV3Net. The modified SqueezeNet achieved 98% accuracy, while the InceptionV3Net classifier achieved 98.4% accuracy.

## 3. Materials and Methods

In this section, we have presented the details of the used dataset and the proposed Attention TurkerNeXt.

### 3.1. Dataset

This research was conducted on a study group consisting of individuals diagnosed with bipolar disorder who were receiving treatment at Elazig Fethi Sekin City Hospital and Elazig Mental and Neurological Diseases Hospital. During the data collection process, the Huvitz HOCT-1F OCT device was utilized. OCT image datasets of bipolar patients were meticulously obtained by scanning the Retinal Nerve Fiber Layer (RNFL) and macular regions. These regions were scanned horizontally to create cross-sectional slices at specified intervals. The study included 20 patients diagnosed with bipolar disorder and 30 healthy control individuals. Bipolar patients were assessed using the Beck Depression Inventory (BDI) and the Young Mania Rating Scale (YMRS). The relevant socio-demographic characteristics of the participants are systematically presented in Table 1.

**Figure 1:**
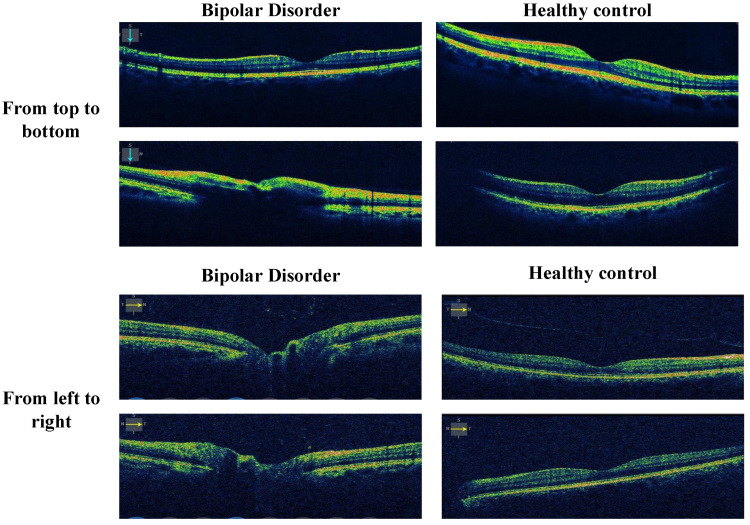
OCT images from the collected dataset corresponding to various cases.

The sample images are given in Figure 1.

In Figure 1, we present the collected sample OCT images with two cases. To verify the hypothesis of nerve-related impairment in the eyes of patients with bipolar disorder using artificial intelligence, a new OCT dataset was assembled. This dataset was sourced from both individuals diagnosed with bipolar disorder and healthy controls. Two types of eye movements were induced to obtain OCT images from these individuals, denoted as vertical (from bottom to top) and horizontal (from left to right) movements. As illustrated in Figure 1, structural differences were observed between OCT images of individuals with bipolar disorder and those of healthy individuals.

The dataset is categorized into two main groups: ‘From left to right’ and ‘From top to bottom.’ In the ‘From left to right’ category, there are a total of 403 images for bipolar disorder and 912 images for healthy controls. Similarly, in the ‘From top to bottom’ category, there are 403 images for bipolar disorder and 912 images for healthy controls. In total, this dataset comprises 2630 images. The allocation of these OCT (optical coherence tomography) images into different training and test sets is succinctly summarized in Table 2.

### 3.2. Attention TurkerNeXt

In this study, we introduced a novel Attention CNN named Attention TurkerNeXt. Our primary goal was to develop a new generation of ConvNeXt. To achieve this, we integrated components from Swin Transformers [32], ConvNeXt [33], MLP [34], and ResNet [35] into our model. We utilized four essential blocks to structure this model, which are detailed below to elucidate the design of the proposed Attention TurkerNeXt.

*Stem block:* In this block, we have used patchify to generate the first feature map. We have used 4 × 4 sized convolution, batch normalization, and swish activation functions in this block. The mathematical definition of this block is given below.

(1) $$out^{1}=swish\left(B\left(C\left(I\right)\right)\right)$$

(2) $$swish\left(x\right)=\frac{xe^{x}}{e^{x}+1}=x\times sigmoid(x)$$

Herein, $out^{1}$ is the first output, B(.) defines the batch normalization function, and C(.) implies convolution function. We have used a 4 × 4 sized stride to create a patchify model.

**Figure 2:**
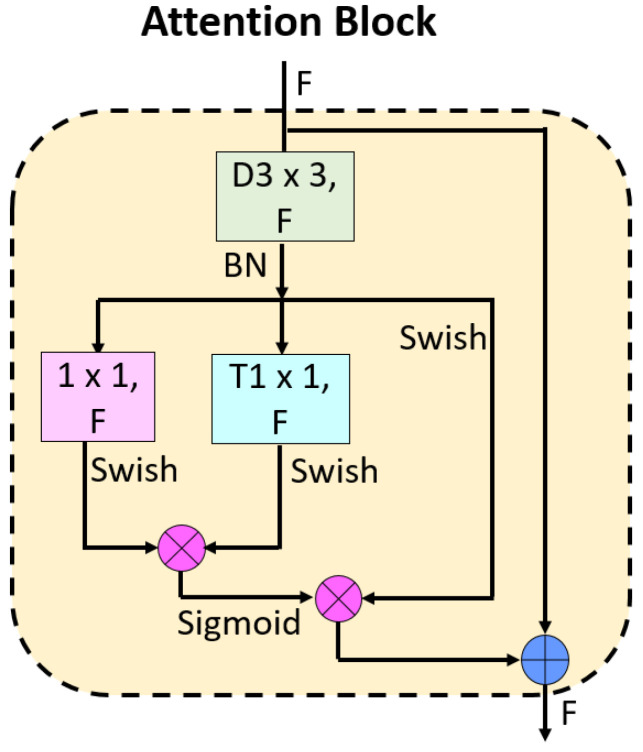
The proposed attention block. Herein, F defines the number of filters, D represents depth-wise convolution, and T is the transposed convolution.

*The proposed attention block (Attention TurkerNeXt block):* The novelty of this model is the proposed attention block. In order to propose this block, we used an MLP structure and shortcut together. In this block, depth-wise convolution, point-wise convolution, and transposed convolution have been used together. A schematic diagram of this block is demonstrated in Figure 2 to clarify this block better.

The mathematical definition of this block is also given below.

(3) $$dout=B\left(C\left(out^{n-1}\right)\right)$$

(4) $$mout=swish\left(C\left(dout\right)\right)\times swish\left(T\left(dout\right)\right)$$

(5) $$at=sigmoid(mout)\times swish\left(dout\right)$$

(6) $$out^{n}=at+out^{n-1}$$

Herein, dout: depth-wise outcome, T(): transposed convolution, mout: multiplication outcome, and at: attention-based output. As can be seen from Figure 1 and the given equations, we have used both attention and residual connections together in this block.

*Downsampling block:* In the downsampling block, we used a 2 × 2 sized convolution with 2 × 2 stride.

*Output block:* To gather outputs, we used global average pooling a fully connected layer, and a softmax function in this block.

**Figure 3:**
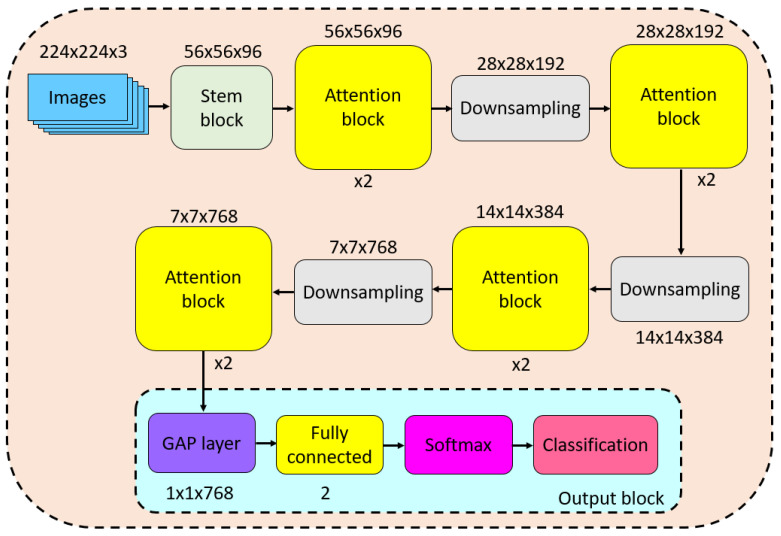
Graphical output of the proposed Attention TurkerNeXt.

By using these blocks, we created the proposed Attention TurkerNeXt, and a block diagram of this ConvNeXt is illustrated in Figure 3.

The graphical output of Attention TurkerNeXt is visually presented in Figure 3, illustrating the interconnected blocks, their sizes, and repetitions.

Per Figure 3, we have presented the transition table of the proposed Attention TurkerNeXt in Table 3.

The novel architecture of Attention TurkerNeXt represents a fusion of cutting-edge neural network components, meticulously designed to excel in the complex task of identifying biomarkers associated with bipolar disorder. The model’s success, as evidenced by its performance metrics, positions it as a pioneering tool in the realm of medical image analysis.

## 4. Experimental Results and Discussions

The results obtained are presented in this section. To implement our proposed model, we utilized the MATLAB 2023a programming environment. The computations were conducted on a personal computer (PC) with 64 GB of main memory, a central processor operating at 3.6 GHz, and a graphics card boasting 1920 CUDA cores. We designed the proposed Attention TurkerNeXt using MATLAB’s deep network designer, building the network from scratch.

The TurkerNeXt was trained on the two cases present in our dataset. The training options for our proposed CNN were as follows:

Solver: Stochastic gradient descent with momentum

Initial learning rate: 0.01

Mini batch size: 32

Number of epochs: 50

Learning drop factor: 0.1

L2 regularization: 0.0001

Momentum: 0.9

Training and validation split ratio: 80:20

**Figure 4:**
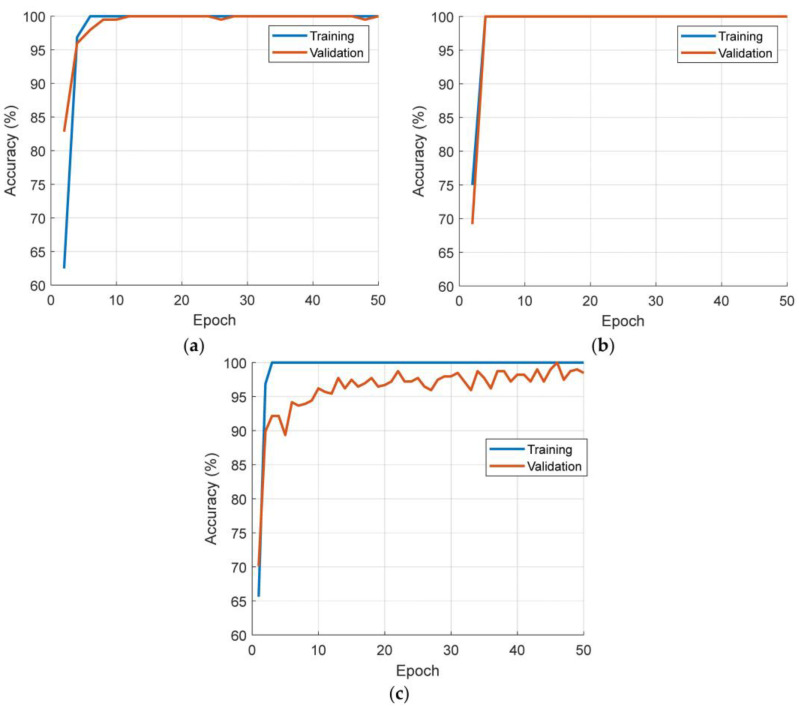
Training and validation curves of the proposed Attention TurkerNeXt. (**a**) Bottom to top. (**b**) Left to right. (**c**) Merged case: bottom to top + left to right.

Each case was trained using the parameters mentioned above, and the resulting training and validation curves are detailed in Figure 4.

**Figure 5:**
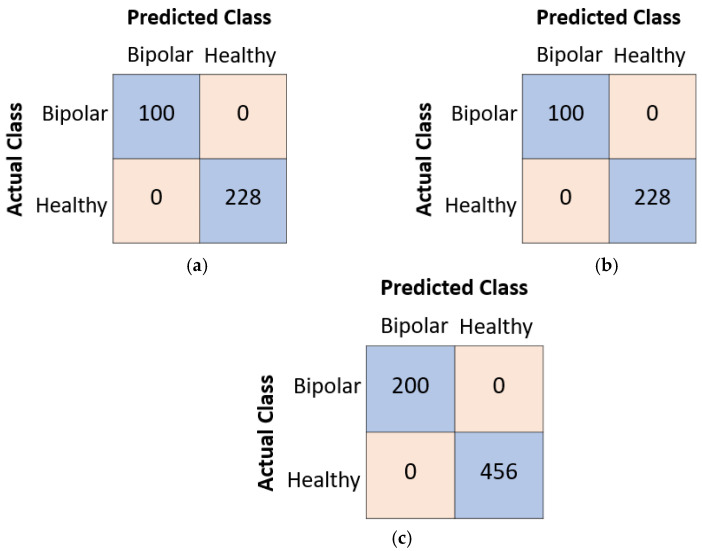
The computed test confusion matrices. (**a**) Bottom to top. (**b**) Left to right. (**c**) Merged.

As shown in Figure 4, our proposed model achieved 100% accuracy in training and validation for all three defined cases. Additionally, we calculated the testing accuracies for these cases, and the confusion matrices for each case are depicted in Figure 5.

As illustrated in Figure 5, the proposed Attention TurkerNeXt achieved 100% accuracy for all three cases. Moreover, we have attained 100% sensitivity, specificity, F1-score, geometric mean and precision. We have listed our results in Table 4.

### 4.1. Explainable Results

**Figure 6:**
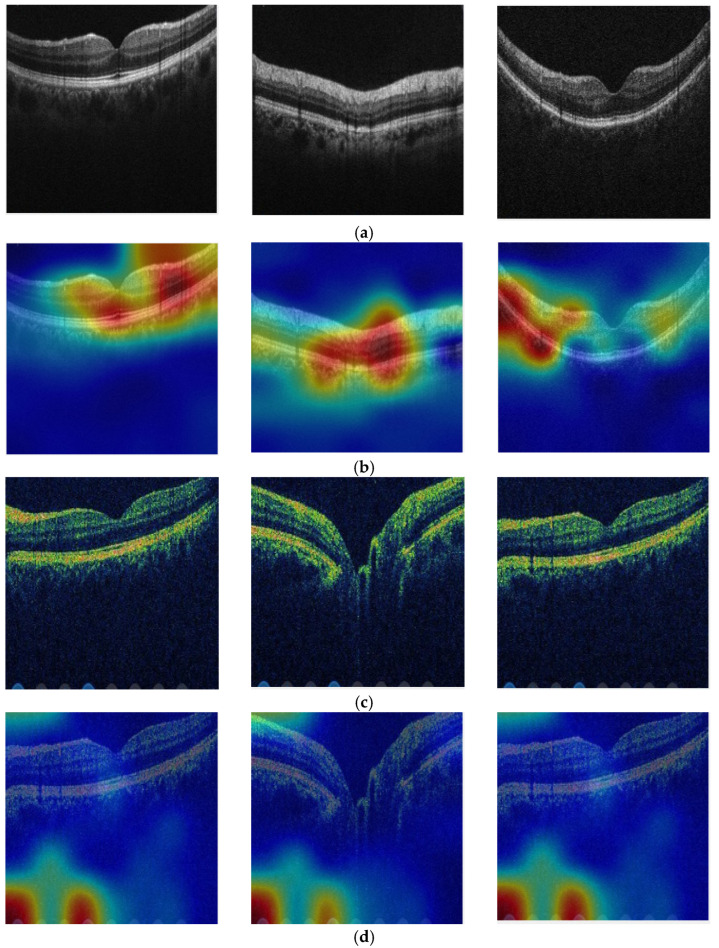
Heatmaps of the used OCT images. (**a**) Healthy OCT images. (**b**) Heatmaps of the healthy OCT images. (**c**) Bipolar OCT images. (**d**) Heatmaps of the bipolar OCT images.

We have presented the explainable results of our proposed model using the collected dataset. To achieve these explainable results, we utilized activations. Gradient-weighted class activation mapping (Grad-CAM) is a prevalent component in explainable artificial intelligence. Essentially, Grad-CAM provides a heatmap visualization of a specific class label. This label can be user-selected or chosen based on the highest softmax probability via Grad-CAM. The heatmap highlights the most significant regions of interest in the images. Thus, Grad-CAM serves as a valuable method for obtaining interpretable results. The heatmaps generated from sample images are depicted in Figure 6.

According to the heatmaps, TurkerNeXt identified bipolar disorder by analyzing specific regions beneath the OCT images, as it effectively recognizes patterns associated with bipolar disorder in these areas. Conversely, healthy OCTs were identified based on the overall shape of the OCT image. Per the obtained explainable findings, bipolar disorder may affect the retinal nerve fiber layer and the retinal pigment epithelium. Differences occurring in these areas can be detected using OCT in the diagnosis of bipolar disorder.

### 4.2. Discussions

This study identified a potential new biomarker by applying a machine learning model. Consequently, we introduced a novel ConvNeXt called Attention TurkerNeXt. The proposed Attention TurkerNeXt achieved 100% classification accuracy when applied to an OCT image dataset. Additionally, we showcased the explainable outcomes generated by the Attention TurkerNeXt.

Comparative results are provided in Table 5 to highlight the classification prowess of our model.

In our reviewed literature, the field of bipolar disorder detection models is notably underrepresented, highlighting the need for innovative approaches. In response to this gap, we conducted a meticulous comparative analysis, strategically juxtaposing the outcomes of our study with those derived from existing OCT image classification models dedicated to similar diagnostic objectives. This comprehensive assessment serves to shed light on the relative efficacy and performance of our proposed model, Attention TurkerNeXt, within the context of bipolar disorder detection. By presenting a detailed comparison, we aim to contribute valuable insights into the potential advancements our model brings to the forefront of medical image analysis, emphasizing its capability to surpass or complement existing methodologies in this critical domain.

**Figure 7:**
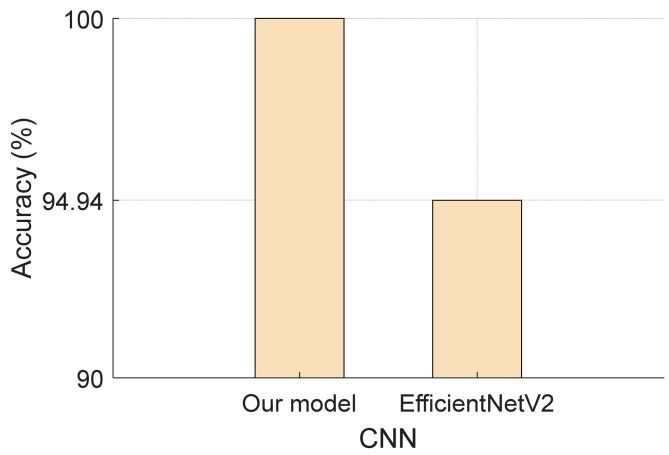
Validation accuracies of the proposed Attention TurkerNeXt and EfficientNetV2 for the merged case.

According to these comparative results, our proposed model achieved a 100% classification rate. Additionally, we compared it with EfficientNetV2 [43] using our combined case, and the results are illustrated in Figure 7.

EfficientNetV2, a new generation CNN, stands out as a significant advancement in the field of deep learning architectures. It is designed to achieve high efficiency and accuracy across a wide range of tasks. In our study, we chose EfficientNetV2 as a reference model to assess and compare its performance against our proposed TurkerNeXt.

To conduct a thorough evaluation, both EfficientNetV2 and TurkerNeXt were applied to our meticulously collected dataset. EfficientNetV2 exhibited a commendable 94.94% validation accuracy, showcasing its robust capabilities in image classification tasks.

In contrast, TurkerNeXt, our proposed model, outperformed EfficientNetV2 by achieving remarkable accuracy scores of 100% in both the validation and test sets on the same dataset. This outcome underscores the effectiveness and superiority of TurkerNeXt in capturing intricate patterns and features relevant to bipolar disorder detection within OCT images.

The exceptional performance of TurkerNeXt suggests its potential as a powerful tool for medical image analysis and biomarker identification. The 100% accuracy in validation and test sets highlights its reliability and precision in distinguishing between individuals with bipolar disorder and healthy controls based on OCT images. This compelling evidence positions TurkerNeXt as a promising candidate for further exploration and application in medical diagnostics and related fields.

Our research’s innovations, findings, advantages, and limitations have been explained below.

*Innovations:*

- In this research, we have proposed a new deep learning algorithm and this Algorithm is named TurkerNeXt.
- We have collected a new OCT image dataset to detect bipolar disorder.
- We have shown the explainable results.

*Findings:*

- The proposed Attention TurkerNeXt model efficiently identified potential biomarkers through an OCT image dataset. We have listed the findings of the proposed Attention TurkerNeXt as below.
  ○ Attention TurkerNeXt integrates components from Swin Transformers, ConvNeXt, MLP, and ResNet. This amalgamation is unique and tailored for the specific task of identifying biomarkers associated with bipolar disorder in OCT images. The thoughtful integration of these diverse components contributes to the model’s adaptability and effectiveness in capturing complex patterns.
  ○ The proposed Attention TurkerNeXt block is a novel addition to the architecture. It combines an MLP structure with a shortcut, incorporating depth-wise convolution, point-wise convolution, and transposed convolution simultaneously. The utilization of attention and residual connections within this block enhances the model’s capacity to capture intricate features relevant to bipolar disorder. This block’s architecture is distinct from traditional CNN building blocks, making it a novel contribution to the field.
  ○ Attention TurkerNeXt stands out by providing explainable results, a critical feature in medical applications. The attention mechanisms integrated into the model contribute to its interpretability, allowing clinicians and researchers to understand the basis for the model’s predictions. This emphasis on explainability is a novel and crucial aspect, especially in the context of medical image analysis.
  ○ The use of the patchify approach in the Stem block to generate the first feature map is a novel strategy. This method, employing a 4 × 4-sized convolution, batch normalization, and swish activation functions, contributes to the model’s initial feature extraction and sets the stage for subsequent processing.
  ○ The graphical output of Attention TurkerNeXt, along with the presented transition table, provides a comprehensive view of the model’s architecture. This transparency is crucial in understanding the flow of information through different layers, contributing to the novelty of the model’s design.
  ○ Achieving a 100% classification accuracy on both the validation and test sets for bipolar disorder detection in OCT images is a remarkable and novel accomplishment. This level of accuracy is indicative of the model’s ability to discern subtle patterns and features associated with bipolar disorder, setting it apart from existing models.

In conclusion, the Attention TurkerNeXt CNN introduces novel elements, from its integrated components to the attention-based block, explainable results, and innovative architectural choices. These aspects collectively contribute to the model’s uniqueness and efficacy in the specific task of identifying biomarkers related to bipolar disorder in OCT images.

- A comparative analysis revealed that the Attention TurkerNeXt outperformed EfficientNetV2, achieving a validation accuracy of 94.94%.

*Advantages:*

- The proposed model achieves perfect classification performance, indicating its reliability and robustness.
- The model does not just provide outcomes; it gives explainable results, enabling better understanding and trustworthiness.
- Compared to existing models like EfficientNetV2, Attention TurkerNeXt showcases superior classification capability, especially in the context of the collected dataset.
- The model’s ability to identify new potential biomarkers can greatly enhance diagnostic methods in medical research.
- The proposed CNN has only 1.6 million parameters. Therefore, the proposed Attention TurkerNeXt is a lightweight model.

*Limitations:*

- Larger and more diverse OCT datasets can be gathered. OCT images from other macular degenerative disorders can be employed to identify patterns indicative of bipolar disorder.
- Attention TurkerNeXt can be tested for other computer vision problems.

## 5. Conclusions

Our research on OCT imaging has made a contribution that has fundamentally reshaped the landscape of bipolar disorder diagnosis. Attention TurkerNeXt, a ConvNeXt model designed for the identification of potential biomarkers associated with bipolar disorder, has emerged as a leading force, not only achieving an outstanding classification accuracy of 100%, but also exceeding the performance of the benchmark model. EfficientNetV2 achieved a 94.94% verification accuracy.

Our proposed model is both a lightweight algorithm and has a high ability to produce explainable results. This critical feature provides an important bridge between machine-driven precision and meaningful interpretation in the complex field of medical diagnosis. Reaching 100% accuracy is not just a numerical milestone; It is proof of the concrete impact and reliability that our model can provide to the front lines of the healthcare industry.

Future efforts should include more comprehensive comparative studies, comprehensive examination of diverse datasets, and rigorous reduction of potential overfitting. While this work is groundbreaking, it also serves as a dynamic foundation for ongoing research and continuous improvement.

## Figures and Tables

**Table 1:** Characteristics of the collected dataset.

Diagnosis	Bipolar Disorder		Healthy Control	
**Sex**	10 female	10 male	15 female	15 male
**Mean age, years**	36.5 ± 4.25	40.4 ± 8.8	28.7 ± 5.36	30.4 ± 3.55
**Age range, years**	25–59	18–62	23–48	27–56
**Beck Depression Inventory (BDI)**	4.37 ± 3.15	4.66 ± 2.87	-	-
**Young Mania Rating Scale (YMRS)**	2 ± 1.51	2.88 ± 4.62	-	-

**Table 2:** Training and test image distributions.

	From Left to Right			From Top to Bottom		
	Train Images	Test Images	Total	Train Images	Test Images	Total
**Bipolar Disorder**	303	100	403	303	100	403
**Healthy control**	684	228	912	684	228	912

**Table 3:** Transition table of the proposed Attention TurkerNeXt.

Layer	Input Size	Operation	Output Size
**Stem**	224 × 224	4 × 4, 96, stride: 4	56 × 56
**Layer 1**	56 × 56	$\left[\begin{array}{c} d3\times 3,\;96\\ 1\times 1,\;96\;⨂\;t1\times 1,\;96 \end{array}\right]\times 2$	28 × 28
**Layer 2**	28 × 28	$\left[\begin{array}{c} d3\times 3,\;192\\ 1\times 1,\;192\;⨂\;t1\times 1,\;192 \end{array}\right]\times 2$	14 × 14
**Layer 3**	14 × 14	$\left[\begin{array}{c} d3\times 3,\;384\\ 1\times 1,\;384\;⨂\;t1\times 1,\;384 \end{array}\right]\times 2$	7 × 7
**Layer 4**	7 × 7	$\left[\begin{array}{c} d3\times 3,\;768\\ 1\times 1,\;768\;⨂\;t1\times 1,\;768 \end{array}\right]\times 2$	7 × 7
**Output size**	7 × 7	Global average pooling, fully connected layer, softmax	Number of classes
**Total learnable parameters**			~1.6 million

**Table 4:** Classification results of the presented Attention TurkerNeXt according to the used cases.

Performance Evaluation Metrics	Case		
	Bottom to Top	Left to Right	Merged
Accuracy	100%	100%	100%
Sensitivity	100%	100%	100%
Specificity	100%	100%	100%
Precision	100%	100%	100%
F1-score	100%	100%	100%
Geometric mean	100%	100%	100%

**Table 5:** Comparative results.

Study	Model	Dataset	Results (%)
[36]	Joint-Attention Network MobileNet-v2	OCT2017 500 training images 500 testing images	Accuracy: 95.60 Specificity: 97.10 Sensitivity: 95.60
	Joint-Attention Network ResNet50-v1	Srinivasan2014 2916 training images 315 testing images	Accuracy: 100.0 Specificity: 100.0 Sensitivity: 100.0
[37]	CNN	16,896 images 100:1	Accuracy: 94.35
[38]	Transfer learning, Ant colony optimization	2397 training images 601 testing images	Accuracy: 99.10
[39]	Swin-Poly Transformer network	OCT-C8 25,600 training images 2800 validation images 2800 testing images	Accuracy: 97.12 Precision: 97.13 Recall: 97.13 F1-Score: 97.10
[40]	Lesion-aware convolution neural network	2000 images 10-fold CV	Accuracy: 90.10 Sensitivity: 86.80 Precision: 86.20
[41]	Hybrid ConvNet–Transformer Network	Srinivasan2014 3231 images 60:20:20	Accuracy: 86.18 Sensitivity:85.40 Precision: 88.53
		OCT2017 84.484 images 60:20:20	Accuracy: 91.56 Sensitivity:88.57 Precision: 88.11
[42]	CNN, iterative ReliefF	Srinivasan2014 3194 images 10-fold CV	Accuracy: 100.0 Precision: 100.0 F1-score: 100.0
		OCT image dataset 11,000 images 10:1	Accuracy: 97.30 Precision: 97.32 F1-score: 97.30
Proposed Model	Attention TurkerNeXt	Collected Dataset 2630 images 60:15:25	**From left to right** Accuracy: 100.0 Sensitivity: 100.0 Specificity: 100.0 **From top to bottom** Accuracy: 100.0 Sensitivity: 100.0 Specificity: 100.0 **Merged** Accuracy: 100.0 Sensitivity: 100.0 Specificity: 100.0

## Data Availability

The Bipolar Disorder Detection OCT Dataset is available upon request.

## References

[1] Cotrena C., Branco L.D., Kochhann R., Shansis F.M., Fonseca R.P. (2016). Quality of life, functioning and cognition in bipolar disorder and major depression: A latent profile analysis. Psychiatry Res..

[2] Uher R. (2014). Gene–environment interactions in severe mental illness. Front. Psychiatry.

[3] Berk M., Berk L., Dodd S., Cotton S., Macneil C., Daglas R., Conus P., Bechdolf A., Moylan S., Malhi G.S. (2014). Stage managing bipolar disorder. Bipolar Disord..

[4] Berk M., Kapczinski F., Andreazza A.C., Dean O.M., Giorlando F., Maes M., Yücel M., Gama C.S., Dodd S., Dean B. (2011). Pathways underlying neuroprogression in bipolar disorder: Focus on inflammation, oxidative stress and neurotrophic factors. Neurosci. Biobehav. Rev..

[5] Anderson G., Maes M. (2015). Bipolar disorder: Role of immune-inflammatory cytokines, oxidative and nitrosative stress and tryptophan catabolites. Curr. Psychiatry Rep..

[6] Andreazza A.C., Young L.T. (2014). The neurobiology of bipolar disorder: Identifying targets for specific agents and synergies for combination treatment. Int. J. Neuropsychopharmacol..

[7] Duong A., Syed B., Scola G. (2015). Biomarkers for bipolar disorder: Current insights. Curr. Biomark. Find..

[8] Scola G., Andreazza A.C. (2014). Current state of biomarkers in bipolar disorder. Curr. Psychiatry Rep..

[9] Singh I., Rose N. (2009). Biomarkers in psychiatry. Nature.

[10] Kempton M.J., Geddes J.R., Ettinger U., Williams S.C., Grasby P.M. (2008). Meta-analysis, database, and meta-regression of 98 structural imaging studies in bipolar disorder. Arch. Gen. Psychiatry.

[11] Vita A., De Peri L., Sacchetti E. (2009). Gray matter, white matter, brain, and intracranial volumes in first-episode bipolar disorder: A meta-analysis of magnetic resonance imaging studies. Bipolar Disord..

[12] Moorhead T.W.J., McKirdy J., Sussmann J.E., Hall J., Lawrie S.M., Johnstone E.C., McIntosh A.M. (2007). Progressive gray matter loss in patients with bipolar disorder. Biol. Psychiatry.

[13] Frey B.N., Zunta-Soares G.B., Caetano S.C., Nicoletti M.A., Hatch J.P., Brambilla P., Mallinger A.G., Soares J.C. (2008). Illness duration and total brain gray matter in bipolar disorder: Evidence for neurodegeneration?. Eur. Neuropsychopharmacol..

[14] Papiol S., Molina V., Desco M., Rosa A., Reig S., Sanz J., Palomo T., Fananas L. (2008). Gray matter deficits in bipolar disorder are associated with genetic variability at interleukin-1 beta gene (2q13). Genes Brain Behav..

[15] Ladouceur C.D., Almeida J.R., Birmaher B., Axelson D.A., Nau S., Kalas C., Monk K., Kupfer D.J., Phillips M.L. (2008). Subcortical gray matter volume abnormalities in healthy bipolar offspring: Potential neuroanatomical risk marker for bipolar disorder?. J. Am. Acad. Child Adolesc. Psychiatry.

[16] Chu E.M.-Y., Kolappan M., Barnes T.R., Joyce E.M., Ron M.A. (2012). A window into the brain: An in vivo study of the retina in schizophrenia using optical coherence tomography. Psychiatry Res. Neuroimaging.

[17] Yeap S., Kelly S.P., Sehatpour P., Magno E., Garavan H., Thakore J.H., Foxe J.J. (2008). Visual sensory processing deficits in Schizophrenia and their relationship to disease state. Eur. Arch. Psychiatry Clin. Neurosci..

[18] Tasci G., Gun M.V., Keles T., Tasci B., Barua P.D., Tasci I., Dogan S., Baygin M., Palmer E.E., Tuncer T. (2023). QLBP: Dynamic patterns-based feature extraction functions for automatic detection of mental health and cognitive conditions using EEG signals. Chaos Solitons Fractals.

[19] Tasci G., Loh H.W., Barua P.D., Baygin M., Tasci B., Dogan S., Tuncer T., Palmer E.E., Tan R.-S., Acharya U.R. (2023). Automated accurate detection of depression using twin Pascal’s triangles lattice pattern with EEG Signals. Knowl.-Based Syst..

[20] Şuheda K., TASCİ B. (2023). Electroencephalogram-Based Major Depressive Disorder Classification Using Convolutional Neural Network and Transfer Learning. Turk. J. Sci. Technol..

[21] Tasci B., Tasci G., Dogan S., Tuncer T. (2022). A novel ternary pattern-based automatic psychiatric disorders classification using ECG signals. Cogn. Neurodyn..

[22] Tatli S., Macin G., Tasci I., Tasci B., Barua P.D., Baygin M., Tuncer T., Dogan S., Ciaccio E.J., Acharya U.R. (2024). Transfer-transfer model with MSNet: An automated accurate multiple sclerosis and myelitis detection system. Expert Syst. Appl..

[23] Tasci B., Tasci G., Ayyildiz H., Kamath A.P., Barua P.D., Tuncer T., Dogan S., Ciaccio E.J., Chakraborty S., Acharya U.R. (2023). Automated schizophrenia detection model using blood sample scattergram images and local binary pattern. Multimed. Tools Appl..

[24] Tasci I., Tasci B., Barua P.D., Dogan S., Tuncer T., Palmer E.E., Fujita H., Acharya U.R. (2023). Epilepsy detection in 121 patient populations using hypercube pattern from EEG signals. Inf. Fusion.

[25] Thomas A., Sunija A.P., Manoj R., Ramachandran R., Ramachandran S., Varun P.G., Palanisamy P. (2021). RPE layer detection and baseline estimation using statistical methods and randomization for classification of AMD from retinal OCT. Comput. Methods Programs Biomed..

[26] Perdomo O., Rios H., Rodríguez F.J., Otálora S., Meriaudeau F., Müller H., González F.A. (2019). Classification of diabetes-related retinal diseases using a deep learning approach in optical coherence tomography. Comput. Methods Programs Biomed..

[27] Lee C.S., Baughman D.M., Lee A.Y. (2017). Deep learning is effective for classifying normal versus age-related macular degeneration OCT images. Ophthalmol. Retin..

[28] Perdomo O., Otálora S., González F.A., Meriaudeau F., Müller H. Oct-net: A convolutional network for automatic classification of normal and diabetic macular edema using sd-oct volumes. Proceedings of the 2018 IEEE 15th International Symposium on Biomedical Imaging (ISBI 2018).

[29] Zhang Q., Liu Z., Li J., Liu G. (2020). Identifying diabetic macular edema and other retinal diseases by optical coherence tomography image and multiscale deep learning. Diabetes Metab. Syndr. Obes..

[30] Abdullahi M.M., Chakraborty S., Kaushik P., Sami B.S. Detection of dry and wet age-related macular degeneration using deep learning. Proceedings of the 2nd International Conference on Industry 4.0 and Artificial Intelligence (ICIAI 2021).

[31] Saleh N., Abdel Wahed M., Salaheldin A.M. (2022). Transfer learning-based platform for detecting multi-classification retinal disorders using optical coherence tomography images. Int. J. Imaging Syst. Technol..

[32] Liu Z., Lin Y., Cao Y., Hu H., Wei Y., Zhang Z., Lin S., Guo B. Swin transformer: Hierarchical vision transformer using shifted windows. Proceedings of the IEEE/CVF International Conference on Computer Vision.

[33] Zheng Q., Liu J., Ji Y., Zhang Y., Chen X., Liu B. (2022). Elevated levels of monocyte-lymphocyte ratio and platelet-lymphocyte ratio in adolescents with non-suicidal self-injury. BMC Psychiatry.

[34] Tolstikhin I., Houlsby N., Kolesnikov A., Beyer L., Zhai X., Unterthiner T., Yung J., Keysers D., Uszkoreit J., Lucic M. (2021). MLP-Mixer: An all-MLP Architecture for Vision. arXiv.

[35] He K., Zhang X., Ren S., Sun J. Deep residual learning for image recognition. Proceedings of the IEEE Conference on Computer Vision and Pattern Recognition.

[36] Kamran S.A., Tavakkoli A., Zuckerbrod S.L. Improving robustness using joint attention network for detecting retinal degeneration from optical coherence tomography images. Proceedings of the 2020 IEEE International Conference On Image Processing (ICIP).

[37] Saraiva A.A., Santos D.B.S., Pimentel P.M.C., Sousa J.V.M., Ferreira N., Batista Neto J.E.S., Soares S., Valente A. Classification of optical coherence tomography using convolutional neural networks. Proceedings of the BIOSTEC 2020: 13th International Joint Conference on Biomedical Engineering Systems and Technologies.

[38] Khan A., Pin K., Aziz A., Han J.W., Nam Y. (2023). Optical coherence tomography image classification using hybrid deep learning and ant colony optimization. Sensors.

[39] He J., Wang J., Han Z., Ma J., Wang C., Qi M. (2023). An interpretable transformer network for the retinal disease classification using optical coherence tomography. Sci. Rep..

[40] Fang L., Wang C., Li S., Rabbani H., Chen X., Liu Z. (2019). Attention to lesion: Lesion-aware convolutional neural network for retinal optical coherence tomography image classification. IEEE Trans. Med. Imaging.

[41] Ma Z., Xie Q., Xie P., Fan F., Gao X., Zhu J. (2022). HCTNet: A Hybrid ConvNet-Transformer Network for Retinal Optical Coherence Tomography Image Classification. Biosensors.

[42] Barua P.D., Chan W.Y., Dogan S., Baygin M., Tuncer T., Ciaccio E.J., Islam N., Cheong K.H., Shahid Z.S., Acharya U.R. (2021). Multilevel deep feature generation framework for automated detection of retinal abnormalities using OCT images. Entropy.

[43] Tan M., Le Q. Efficientnetv2: Smaller models and faster training. Proceedings of the International Conference on Machine Learning.

